# Perception of Sour Taste in Subjects with Olfactory Deficits: Role of Myrtle Aromatization

**DOI:** 10.3390/nu17010105

**Published:** 2024-12-30

**Authors:** Antonella Rosa, Paolo Solla, Ilenia Pinna, Francesco Loy, Carla Masala

**Affiliations:** 1 Department of Biomedical Sciences, University of Cagliari, Cittadella Universitaria, SP 8 Monserrato, 09042 Cagliari, Italy; anrosa@unica.it (A.R.); ilenia.pinna@unicam.it (I.P.); floy@unica.it (F.L.); 2 Neurological Unit, AOU Sassari, University of Sassari, Viale S. Pietro 10, 07100 Sassari, Italy; psolla@uniss.it

**Keywords:** sour taste, gustatory function, hyposmia, taste perception

## Abstract

Background: Sour taste is associated with acid-base homeostasis, which is critical to cell metabolism and health conditions. Vinegar, which contains acetic acid as the main component, is a sour food considered the second most common condiment in Italy. Objectives: The aim of the study was to assess differences in sourness perception in subjects with olfactory deficits compared to controls and evaluate myrtle aromatization’s potential effect in modulating sourness perception in subjects with hyposmia. Methods: To this end, olfactory function was assessed with the Sniffin’ Sticks test and gustatory function by the Taste Strips test. Sensory perception of a traditional white wine vinegar (WV) and a WV aromatized with myrtle (AWV) was evaluated. The sourness perception of the two vinegars was estimated through the rates of odor and taste pleasantness, intensity, and familiarity using a labeled hedonic Likert-type scale. Results: Our data indicated that in patients with hyposmia, a significant decrease was observed only in sour taste perception compared to controls. The increase in vinegar aroma due to the myrtle aromatization modulated sourness perception in patients with hyposmia. Conclusions: Myrtle aromatization increased the number of significant correlations between odor and the taste dimensions of the vinegar in controls and in patients with hyposmia in a different manner.

## 1. Introduction

Flavor perception is obtained through gustatory, olfactory, and trigeminal functions. Olfactory and gustatory functions show a bidirectional relationship and are involved in eating behavior, food intake, and food enjoyment [1,2]. The trigeminal function, which plays a critical role in protection from harmful substances, is mediated through trigeminal receptor activation in the oral cavity and nasal mucosa [3,4]. In fact, many odors at a high concentration may stimulate the olfactory system and trigeminal function [4,5]. In humans, significant individual differences were observed in the olfactory and gustatory perception of irritant stimuli. The mechanism of irritant stimuli response has been partially explored due to the close relationship between the olfactory, gustatory, and trigeminal functions.

Following the COVID-19 pandemic, public interest in the role of smell in daily life has increased. Olfactory dysfunction may be related to a decrease in the ability to perceive odors during sniffing (orthonasal olfaction) or during eating and drinking (retronasal olfaction). The decrease in olfactory function is associated with aging, as well as neurodegenerative diseases including Parkinson’ disease (PD).

Olfactory deficits are classified as quantitative dysfunction (such as anosmia and hyposmia) and qualitative dysfunction (such as phantosmia and parosmia) [6]. Patients with quantitative olfactory deficits such as hyposmia, which reflects a decrease in olfactory function, or anosmia, which is a complete olfactory function loss, also showed a decreased taste function [7]. Contradictory data are available as regards the association between olfactory function and flavor perception. Previous studies indicated that subjects with self-reported olfactory deficits showed changes in food preferences [8,9] and taste function [10]. Conversely, another study indicated that patients with congenital anosmia did not show any change in food preference [11]. A decreased taste function may have a negative impact on dietary habits, physical well-being, and quality of life. Previous studies indicated that patients with olfactory dysfunction showed changes in flavor perception related to decreased sensitivity to trigeminal stimuli [12,13,14].

Gustatory stimuli are described considering the five fundamental taste modalities: sweet, sour, salty, bitter, and umami. In particular, the sour taste remains more enigmatic compared to the other taste modalities, and the precise relationships between the olfactory function and sourness in flavor perception are not well known.

Sour taste is induced in humans by organic and inorganic acids. Strong organic acids may induce sourness through protons, while for inorganic acids that are fully dissociated, the sourness is related to pH and the availability of protons. Instead, for partially dissociated organic acids, the sour taste is directly related to free protons and protonated organic acids. Fully ionized strong acid may enter taste cells through the Zn^2+^-sensitive H^+^ channel [15]. Sour taste has been considered the most enigmatic among the five basic taste modalities, since only recently the sour taste receptor Otopetrin1 (OTOP1) was discovered in the type III taste receptor cells of vertebrates [16,17]. OTOP1, which encodes for a novel proton channel in taste buds, is involved in sour taste transduction [18,19], in metabolic homeostasis, and in obesity-induced inflammation [20]. OTOP1 is a selective channel for protons characterized by an inward depolarizing current in response to sour stimuli. Sour taste is strictly associated with acid-base homeostasis in the blood and tissues, which is critical to cell metabolism and health conditions [21].

In the human diet, there are many sour foods such as citrus fruits (lemons, limes, and oranges), yogurt, and vinegar. Vinegar is made by grain or fruit fermentation, which turns sugar into alcohol and creates acetic acid. Acetic acid is considered the main component of vinegar and may induce a sour taste. Vinegar is considered the second most used condiment in Italy, after olive oil [22]. Vinegar is a healthy food with different functional properties, such as antihyperlipidemic effects to prevent cardiovascular disorders and antitumor, antidiabetic, antimicrobial, and anti-inflammatory effects [23,24]. However, acetic acid, which is a weak acid, produces an irritating sensation with the activation of the trigeminal system in the oral and nasal cavities. Exposure to acetic acid induces the activation of nociceptors, which includes acid-sensing ion channels and transient receptor potential channels [25].

In this context, the aim of this study was to evaluate statistical differences in sourness perception in subjects with olfactory deficits in comparison to healthy controls and evaluate the potential effect of aromatic plants in sourness perception modulation in subjects with hyposmia. In our previous studies, we suggested the important role of Mediterranean aromatic plants in salty and bitter perception improvement in patients with hyposmia [26,27].

Firstly, we evaluated differences in sourness perception in patients with pure hyposmia and hyposmia induced by a neurodegenerative disorder (Parkinson’s disease, PD) compared to healthy controls. To this end, olfactory function was assessed with the Sniffin’ Sticks test, and gustatory function (basic taste qualities: sweet, bitter, sour, and salty) was assessed by the Taste Strips test.

Then, differences in the sourness perception of sour stimuli (vinegars) were evaluated in a subpopulation of patients with pure hyposmia compared to age-matched healthy controls. The sensory perception of a traditional white wine vinegar (WV) and a white wine vinegar aromatized with myrtle (AWV) was evaluated to evidence the potential role of this aromatic plant, amply used in the Mediterranean basin for medicinal and culinary purposes [28,29,30], in modulating sourness perception in patients with hyposmia. The sourness perception of the two vinegars was estimated considering odor and taste pleasantness, intensity, and familiarity dimensions using the labeled hedonic Likert-type scale, as previously used in the sensory properties assessment of various food products [26,27,31].

## 2. Materials and Methods

### 2.1. Participants

In this study, 449 subjects were enrolled, which were divided into the following subgroups: 220 healthy subjects with normosmia (mean age ± standard deviation, 33.5 ± 14.3 years), 142 subjects with pure hyposmia (mean age ± standard deviation, 36.3 ± 16.3 years), and 87 patients with PD (mean age ± standard deviation, 69.1 ± 9.9 years). Healthy subjects and patients with hyposmia were enrolled at the University of Cagliari, Italy. PD patients were recruited during regular follow-up visits at the Movement Disorders Center of the University of Sassari (Italy). All participants gave their written informed consent to participate in the study. The PD was diagnosed in accordance with the Movement Disorder Society Clinical Diagnostic Criteria for PD [32] as performed by a neurologist specialized in movement disorders. In all participants, age, height (m), weight (kg), body mass index (BMI, kg/m^2^), smoking status, and gustatory and olfactory function data were collected. The study was conducted in accordance with the Declaration of Helsinki and approved by the Ethical Committee of the University of Cagliari (protocol number: 3605 10/01/24).

### 2.2. Procedures to Evaluate Gustatory Function

The Taste Strips test (Burghart Messtechnik, Wedel, Germany) was performed for the assessment of gustatory function. The Taste Strips test consists of filter paper strips impregnated with four concentrations of each basic taste quality: sour (0.3, 0.165, 0.09, 0.05 g/mL of citric acid), sweet (0.4, 0.2, 0.1, 0.05 g/mL of sucrose), bitter (0.006, 0.0024, 0.0009, 0.0004 g/mL of quinine hydrochloride), and salty (0.25, 0.1, 0.04, 0.016 g/mL of sodium chloride) [33]. The total Taste Strips score, which is the sum of the scores obtained in each taste modality, may range from 0 to 16, and a score < 9 is classified as hypogeusia [33]. Drinking water was used to rinse the participant’s mouth before the test and as a solvent in each taste modality.

### 2.3. Procedures to Evaluate Olfactory and Cognitive Function

The Sniffing’ Sticks test [34,35] was used to evaluate olfactory function, and it consists of three different sub-tasks: odor threshold (OT), odor discrimination (OD), and odor identification (OI). First, the OT was detected using n-butanol with 16 stepwise dilutions. The OT was assessed using a single-staircase technique based on a three-alternative forced-choice task (3AFC). Second, in the OD test three pens were presented, two containing the same odor and the third containing the target odorant using a 3AFC task. Third, OI was evaluated using 16 common odors, each presented with four verbal descriptors in a multiple forced-choice format (three distractors and one target). Total scores (threshold + discrimination + identification = TDI) were calculated. Scores ≤16, between 16.25 and 30.5, between 30.75 and 41.25, and >41.5 were classified as functional anosmia, hyposmia, normosmia, and supersmellers, respectively [36].

Cognitive abilities were evaluated by the Montreal Cognitive Assessment (MoCA), which assesses cognitive function in different domains: visual–spatial skills, executive functions, attention and concentration, memory, language, conceptual thinking (abstraction), calculations, and spatial orientation [37]. The MoCA total score is 30, and any score ≥ 26 was considered normal.

### 2.4. White Wine Vinegar and White Wine Vinegar Aromatized with Myrtle

The WV and the myrtle berry AWV (Table 1) were manufactured and kindly provided by the “Acetificio Remigio Spiga S.N.C.” in Cagliari, Sardinia, Italy. The two types of commercial vinegars were produced using a traditional acetic fermentation of highly selected white wines.

Table 1 indicated nutritional values per 100 mL of the products as reported on the commercial labels.

The WV showed a clear and bright appearance, a straw yellow color, and an intense and pleasantly vinegary aroma.

The aroma of the AWV was characteristic, intense, and pleasantly vinegary, with notes of Mediterranean shrubs, while the taste was pleasantly vinegary with a characteristic myrtle aftertaste.

### 2.5. Procedures to Evaluate Odor and Taste Pleasantness, Intensity, and Familiarity of White Wine Vinegars

From all the participants, a sub-group of subjects (39 with hyposmia and 57 with normosmia) was randomly enrolled to assess the sensory properties of WV and AWV. Non-trained participants were asked to estimate the sensory dimensions of white wine vinegars using a hedonic scale method (self-reported Likert scale) [26,31,38]. For olfactory and gustatory assessments, filter paper strips were impregnated by immersing them in an aliquot (2 mL) of the vinegars. Firstly, the subjects smelled the sample and were asked to indicate the subjective aroma attributes/descriptors that they perceived with more intensity. Then, participants evaluated the odor pleasantness, intensity, and familiarity of the WV and AWV. Participants rinsed their mouths using drinking water prior to the taste experiment. Then, the subjects estimated the taste pleasantness, intensity, and familiarity of the two types of white wine vinegars and produced individual sensory descriptions, such as the presence of a peculiar flavor note. The odor and taste pleasantness, intensity, and familiarity of the two types of white wine vinegars were estimated using a 7-point Likert-type scale, which ranged from 0/not at all to 6 (0 = very unpleasant and 6 = very pleasant; 0 = not intense at all and 6 = very intense; 0 = not familiar at all and 6 = very familiar). A value of 3 was considered a neutral point [26,31,38].

### 2.6. Statistical Analysis

First, a simple size calculation was performed to evaluate the required minimum number of patients in this study. Based on previous similar studies [26,27], a number of around 50 total subjects could be considered adequate to detect significant differences. A power analysis for the one-way ANOVA and bivariate correlation indicated that the minimum sample size to obtain a statistical power of at least 0.8, with an alpha of 0.05 and a medium effect size (d = 0.5), is about 55. Results are indicated as mean values ± standard deviation (SD). Between subjects, one-way ANOVAs and post hoc analyses using Bonferroni’s multiple pairwise comparison test were carried out to evaluate statistical differences in olfactory and gustatory function between patients with pure hyposmia and PD compared to healthy controls. Significant differences in ratings of odor and taste pleasantness, intensity, and familiarity of the two types of white wine vinegars were calculated using a two-way analysis of variance (two-way ANOVA) adjusted with the Bonferroni multiple pairwise comparison tests. Bivariate correlations were calculated using Pearson’s coefficient (r) between olfactory function and sour taste perception in patients with hyposmia, in those with Parkinson’ disease, and in healthy controls. Then, bivariate correlations using Pearson’s coefficient (r) were also performed to evaluate potential correlations between olfactory and gustatory parameters for white vinegar and aromatized white vinegar in patients with hyposmia and in healthy controls. Furthermore, a multivariate linear regression analysis was performed to assess the potential contribution of myrtle aromatization to odor and taste dimensions (pleasantness, intensity, and familiarity) in controls and patients with hyposmia.

All statistical analyses were calculated by SPSS software version 25 for Windows (IBM, Armonk, NY, USA). The significance level was set at *p* < 0.05.

## 3. Results

### 3.1. Differences Between Patients with Hyposmia and Those with Parkinson’ Disease Compared to Healthy Controls for Olfactory and Gustatory Function

In our study, patients with hyposmia and those with PD showed a significant increase in age compared to healthy subjects (Table 2) [F_(2,448)_ = 206.06, *p* < 0.001]. PD patients exhibited a significant increase in age compared to those with hyposmia (*p* < 0.001).

Patients with hyposmia and those with PD exhibited a significant increase in weight [F_(2,448)_ = 13.26, *p* < 0.001] and BMI compared to healthy subjects.

Moreover, patients with PD exhibited an increase in weight (*p* < 0.01) and BMI (*p* < 0.05) compared to those with pure hyposmia. PD patients showed a significant decrease in cognitive function (MoCA score) compared to those with hyposmia and to healthy controls [F_(2,448)_ = 57.5, *p* < 0.001].

**Figure 1:**
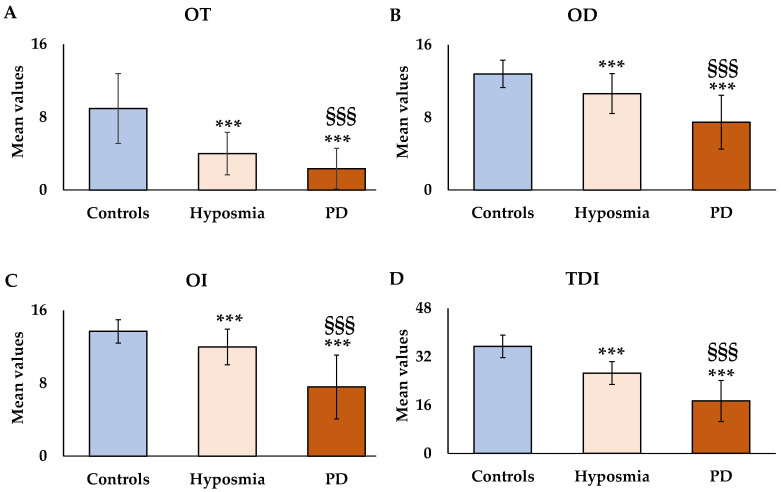
Mean values ± standard deviations of odor threshold (OT) (**A**), odor discrimination (OD) (**B**), odor identification (OI) (**C**), and their sum (TDI score) (**D**) in patients with hyposmia (*n* = 142) and those with Parkinson’s Disease (PD) (*n* = 87) compared to healthy controls (*n* = 220). *** = *p* < 0.001 versus healthy controls; ^§§§^ = *p* < 0.001 between patients with hyposmia and with PD. All statistical differences were established by one-way ANOVA followed by Bonferroni’s post hoc test.

Patients with pure hyposmia and those with PD showed a significant impairment in OT [F_(2,448)_ = 203.75, *p* < 0.001, Figure 1A], in OD [F_(2,448)_ = 208.63, *p* < 0.001, Figure 1B], and in OI [F_(2,448)_ = 264.31, *p* < 0.001, Figure 1C] compared to healthy controls.

Consequently, patients with hyposmia and those with PD showed a significant decrease in global olfactory function (TDI score) compared to healthy controls [F_(2,448)_ = 535.98, *p* < 0.001, Figure 1D].

Moreover, significant differences (*p* < 0.001) were also found between patients with hyposmia and those with PD in OT, OD, OI, and TDI scores. Patients with PD showed a significant impairment in all parameters of olfactory function compared to those with pure hyposmia. Among PD patients, 11.5% (*n* = 10) of them showed anosmia and 88.5% (*n* = 77) exhibited hyposmia.

**Figure 2:**
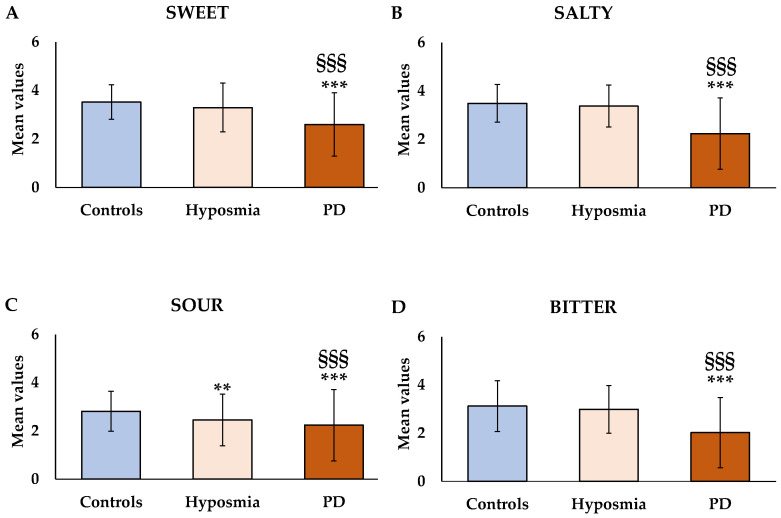
Mean values ± standard deviations of the sweet (**A**), salty (**B**), sour (**C**), and bitter (**D**) taste perception in patients with hyposmia (*n* = 142) and with Parkinson’s Disease (PD) (*n* = 87) compared to healthy controls (*n* = 220). *** = *p* < 0.001, ** = *p* < 0.01 versus healthy controls; ^§§§^ = *p* < 0.001 between patients with hyposmia and with PD. All statistical differences were established by one-way ANOVA followed by Bonferroni’s post hoc test.

As regards gustatory function, we observed a significant decrease in sweet [F_(2,448)_ = 29.99, *p* < 0.001, Figure 2A], salty [F_(2,448)_ = 53.78, *p* < 0.001, Figure 2B], sour [F(_2,448)_ = 42.21, *p* < 0.001, Figure 2C], and bitter [F_(2,448)_ = 31.01, *p* < 0.001, Figure 2D] taste perception in PD patients compared to healthy controls. In addition, PD patients showed a significant decrease (*p* < 0.001) in sweet, salty, sour, and bitter taste perception compared to those with hyposmia.

In patients with pure hyposmia, no significant differences were observed for sweet, salty, and bitter taste perception compared to healthy controls. Interestingly, patients with hyposmia showed a significant decrease (*p* < 0.01) in sour perception compared to healthy controls.

Consequently, patients with hyposmia and those with PD showed a significant decrease in global gustatory function compared to healthy controls [F_(2,448_) = 95.82, *p* < 0.001].

Our data suggested a severe impairment in olfactory and gustatory function in PD patients compared to those with hyposmia. Moreover, PD patients showed a decrease in cognitive function (MoCA mean scores 20.40 ± 6.42), which indicated a mild cognitive deficit, compared to those with hyposmia (27.9 ± 1.67).

### 3.2. Pearson’s Correlations Between Olfactory and Gustatory Function in Patients with Hyposmia, in Those with Parkinson’ Disease, and in Healthy Controls

To evaluate potential associations between demographic parameters and all olfactory/gustatory parameters, Pearson’s correlations (r) were determined in patients with hyposmia, in those with PD, and in healthy controls.

**Figure 3:**
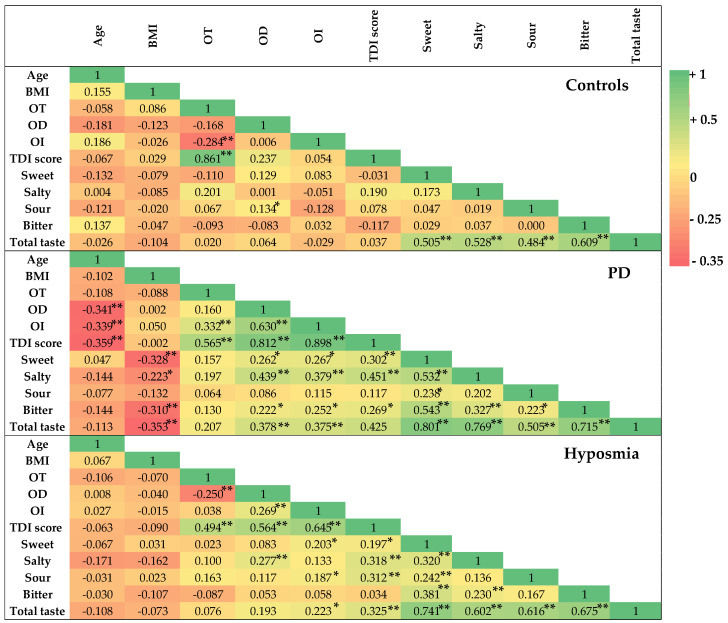
Heatmap of Pearson’s correlations (r) and their significances (** *p* < 0.01; * *p* < 0.05) for age, BMI, olfactory parameters (odor threshold = OT; odor discrimination = OD; odor identification = OI; OT + OD + OI = TDI score), and gustatory parameters (sweet, salty, sour, bitter, and total taste (TT) scores) determined in healthy controls (*n* = 220), in patients with PD (*n* = 87), and in those with hyposmia (*n* = 142).

Figure 3 shows the heatmap of Pearson’s correlations (r) and significance (*p*) calculated between age, BMI, OT, OD, OI, TDI score, sweet, salty, sour, bitter, and TT score in the three groups.

Our data showed that healthy controls exhibited fewer correlations between different olfactory and gustatory parameters than patients with hyposmia and those with PD.

In the controls group, only a weak significant correlation was observed between sour taste perception and OD (r = 0.134, *p* < 0.05).

In PD patients, significant correlations were observed between OD versus sweet, salty, bitter, and total taste (r = 0.262, *p* < 0.05; r = 0.439, *p* < 0.01; r = 0.222, *p* < 0.05; r = 0.378, *p* < 0.01, respectively). Similarly, in PD patients significant correlation were found between OI versus sweet, salty, bitter, and total taste (r = 0.267, *p* < 0.05; r = 0.379, *p* < 0.01; r = 0.252, *p* < 0.05; r = 0.375, *p* < 0.01, respectively) and between TDI score versus sweet, salty, bitter, and total taste (r = 0.302, *p* < 0.01; r = 0.451, *p* < 0.01; r = 0.269, *p* < 0.05; r = 0.425, *p* < 0.01, respectively). Instead, in PD patients no significant correlations were observed between sour taste perception and each parameter of olfactory and gustatory function.

In patients with hyposmia, significant correlations were found between OD versus salty taste (r = 0.277, *p* < 0.01), between OI versus sweet (r = 0.203, *p* < 0.05), and between TDI score versus salty (r = 0.318, *p* < 0.01). Interestingly, in patients with pure hyposmia the sour taste perception was significantly correlated to the OI scores (r = 0.187, *p* < 0.05) and global olfactory function (TDI score) (r = 0.312, *p* < 0.01). Moreover, a significant correlation was observed between sour taste versus sweet perception (r = 0.242, *p* < 0.01).

**Figure 4:**
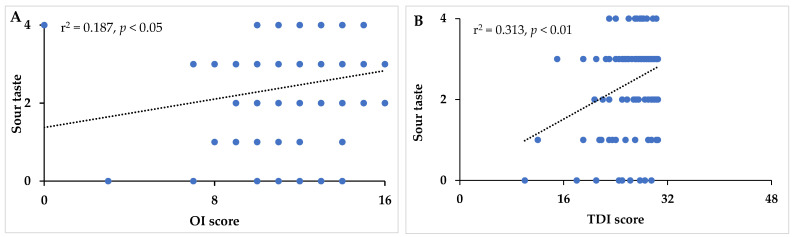
Scatterplots of the Pearson correlations calculated between sour taste perception versus odor intensity (OI) (**A**) and TDI score (**B**) in patients with hyposmia.

The scatterplots with the Pearson correlations, calculated between sour taste perception versus OI and TDI scores in patients with hyposmia, are reported in Figure 4A and Figure 4B, respectively.

### 3.3. Ratings of Odor and Taste Pleasantness, Intensity, and Familiarity for White Wine Vinegars

Our data showed that patients with pure hyposmia exhibited a significant impairment in sour taste perception without any cognitive decline.

Consequently, we focused our attention on a subpopulation of patients with pure hyposmia compared to healthy controls, to evaluate the sensory perception of vinegar and the potential role of myrtle aromatization in modulating the vinegar odor and taste dimensions.

A subpopulation of 96 subjects (39 patients with pure hyposmia and 57 age-matched healthy controls) was selected.

Demographic and clinical parameters of two groups are reported in Table 3. No significant differences are shown between patients with hyposmia compared to healthy controls for age, weight, height, and BMI.

In this subpopulation, the olfactory and gustatory function in patients with hyposmia and healthy controls were similar to those of the same groups in the total population.

**Figure 5:**
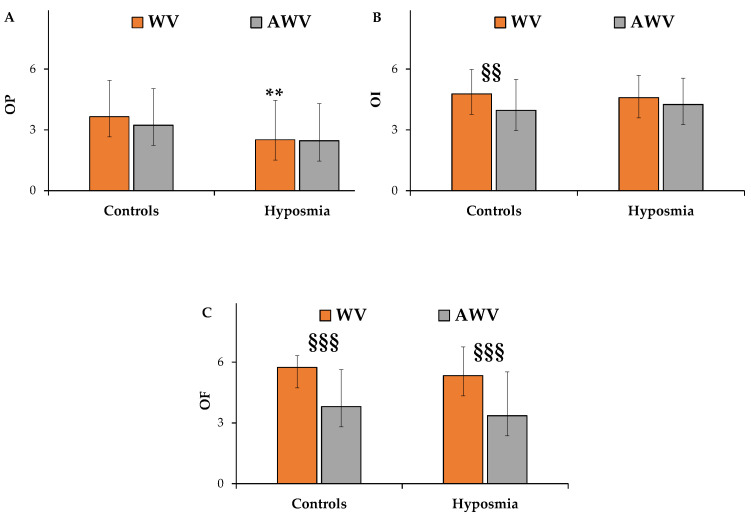
Ratings of odor pleasantness (OP) (**A**), intensity (OI) (**B**), and familiarity (OF) (**C**) dimensions of white wine vinegar (WV) and aromatized white wine vinegar (AWV) measured in healthy controls (*n* = 57) and patients with hyposmia (*n* = 39). Results are expressed as mean values ± standard deviations; ** = *p* < 0.01 between patients with hyposmia compared to healthy controls; ^§§^ = *p* < 0.01 and ^§§§^ = *p* < 0.001. All statistical differences were established by two-way ANOVA followed by Bonferroni’s post hoc test.

Figure 5A showed the ratings of odor pleasantness for the two types of white wine vinegars in patients with hyposmia compared to healthy controls.

Generally, patients with hyposmia perceived both vinegar odors as less pleasant than healthy controls.

Regarding traditional WV, patients with hyposmia exhibited a significant decrease in odor pleasantness [F_(1,188)_ = 12.58, *p* < 0.01] compared to controls. Mean values ± standard deviation for WV were 3.65 ± 1.79 and 2.51 ± 1.94, in controls and in patients with hyposmia, respectively.

The myrtle aromatization induced a slight decrease in odor pleasantness in healthy controls, while no evident decrease was observed between AWV and WV in patients with hyposmia.

Figure 5B reports ratings of odor intensity for the two types of vinegars in patients with hyposmia compared to healthy controls.

Similar mean values of odor intensity for WV were observed in patients with hyposmia and controls.

In the control group, AWV was perceived as significantly less intense than WV [F_(1,188)_ = 8.85, *p* < 0.01]. The mean values ± standard deviation of odor intensity in controls were 4.77 ± 1.21 and 3.96 ± 1.52 for WV and AWV, respectively.

In patients with hyposmia, no significant differences for odor intensity were found between the two types of vinegars, suggesting a potential role of myrtle aromatization in the modulation of WV intensity perception. The mean values ± standard deviation of odor intensity in patients with hyposmia were 4.59 ± 1.09 and 4.26 ± 1.29 for WV and AWV, respectively.

Aromatized AWV was perceived as less familiar than the WV [F_(1,188)_ = 72.25, *p* < 0.001] both in controls and in patients with hyposmia [F_(1,188)_ = 8.56, *p* < 0.001] (Figure 5C).

In controls, the mean values ± standard deviation of odor familiarity were 5.74 ± 0.58 and 3.81 ± 1.82 for WV and AWV, respectively. In patients with hyposmia, the mean values ± standard deviation were 5.33 ± 1.42 and 3.36 ± 2.16 for WV and AWV, respectively.

**Figure 6:**
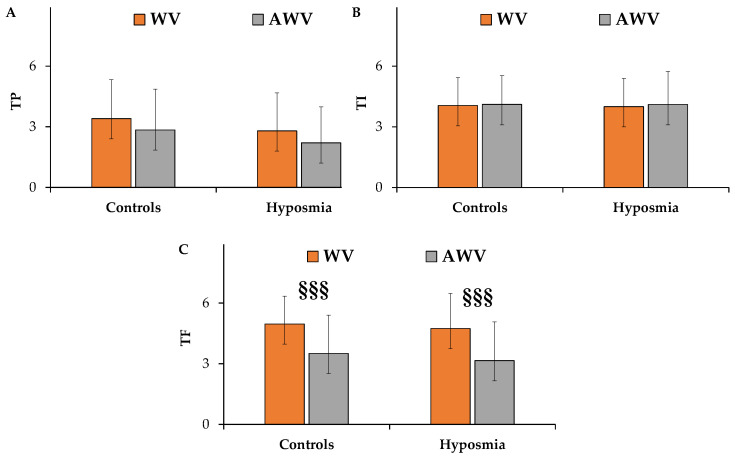
Ratings of taste pleasantness (TP) (**A**), intensity (TI) (**B**), and familiarity (TF) (**C**) dimensions of white wine vinegar (WV) and aromatized white wine vinegar (AWV) measured in healthy controls (*n* = 57) and patients with hyposmia (*n* = 39). Results are expressed as mean values ± standard deviations. ^§§§^ = *p* < 0.001 between white wine vinegar and aromatized white wine vinegar. All statistical differences were established by two-way ANOVA followed by Bonferroni’s post hoc test.

Figure 6 explains the ratings of taste pleasantness, intensity, and familiarity of WV and AWV in patients with hyposmia compared to healthy controls.

Generally, patients with hyposmia perceived both vinegar tastes as less pleasant than healthy controls (Figure 6A).

The myrtle aromatization induced a slight decrease in taste pleasantness both in controls and in patients with hyposmia.

Regarding WV, patients with hyposmia showed similar ratings of taste intensity compared to healthy controls (Figure 6B). The WV taste intensity was principally identified with the intensity of its sourness.

The myrtle aromatization did not induce a change in vinegar taste intensity in both groups. Similar mean values of taste familiarity were observed for WV in patients with hyposmia (5.33 ± 1.42) and controls (5.74 ± 0.58) (Figure 6C). All subjects perceived the AWV taste as significantly (*p* < 0.001) less familiar than that of the WV.

**Figure 7:**
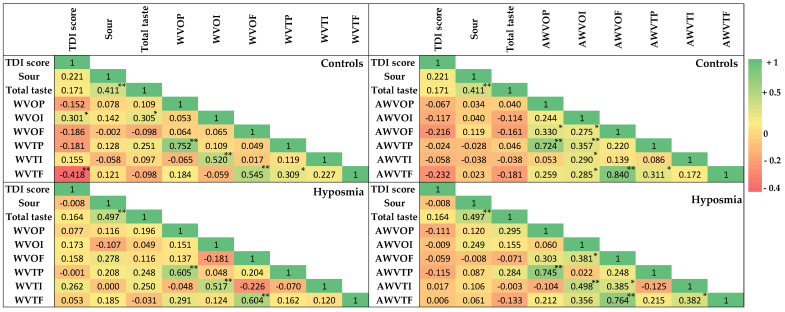
Heatmap of Pearson’s correlations (r) and their significances (** *p* < 0.01; * *p* < 0.05) for TDI score, sour taste, bitterness, odor pleasantness, odor intensity, odor familiarity, taste pleasantness, taste intensity, and taste familiarity of white wine vinegar (WVOP, WVOI, WVOF, WVTP, WVTI, and WVTF, respectively) and aromatized white wine vinegar (AWVOP, AWVOI, AWVOF, AWVTP, AWVTI, and AWVTF, respectively) in healthy controls (*n* = 57) and in patients with hyposmia (*n* = 39).

Figure 7 indicates Pearson’s correlations (r) of olfactory and gustatory parameters for WV and AWV in patients with hyposmia and in healthy controls.

In healthy controls, tight correlations were found between the WV and AWV odor and taste dimensions. As regards traditional WV, significant correlations were found between TDI score versus the odor pleasantness (r = 0.301, *p* < 0.05) and versus odor familiarity (r = −0.418, *p* < 0.01). In addition, for traditional white wine vinegar we also observed significant correlations, between odor pleasantness versus taste pleasantness (r = 0.752, *p* < 0.01), odor intensity versus total taste function (r = 0.305, *p* < 0.05), odor intensity versus taste intensity (r = 0.520, *p* < 0.01), odor familiarity versus taste familiarity (r = 0.545, *p* < 0.01), and taste pleasantness versus taste familiarity (r = 0.309, *p* < 0.05). No significant correlations were found between sour taste perception and any other WV and AWV odor and taste dimensions.

As regards AWV, the following significant correlations were shown: between odor pleasantness versus taste pleasantness (r = 0.724, *p* < 0.01); between odor intensity versus taste intensity (r = 0.290, *p* < 0.05), taste familiarity (r = 0.285, *p* < 0.05), and taste pleasantness (r = 0.357, *p* < 0.01); and between odor familiarity versus taste familiarity (r = 0.840, *p* < 0.01).

In patients with hyposmia, significant correlations were found for the WV and AWV odor and taste dimensions. No significant correlations were observed between TDI score versus WV odor intensity and between sour taste versus any other odor or taste dimension. As regards WV, odor pleasantness was correlated to taste pleasantness (r = 0.605, *p* < 0.01), odor intensity to taste intensity (r = 0.517, *p* < 0.01), and odor familiarity to taste familiarity (r = 0.604, *p* < 0.01). For the myrtle aromatized WV, odor pleasant was correlated versus taste pleasant (r = 0.745, *p* < 0.01), odor intensity versus taste intensity (r = 0.498, *p* < 0.01), odor familiarity versus taste familiarity (r = 0.764, *p* < 0.01).

Our data indicated that myrtle aromatization increased the number of correlations between the odor and taste dimensions of the white wine vinegar both in controls and in patients with hyposmia in a different manner. To investigate the potential role of myrtle aromatization on odor and taste dimensions, we performed multiple linear regression analyses in controls and patients with hyposmia, using AWV odor intensity as a dependent variable.

Table 4 shows multiple regression analyses performed for the odor and taste dimensions (pleasantness, intensity, and familiarity) of AWV in healthy controls (Table 4A) and patients with hyposmia (Table 4B).

In controls, AWV odor intensity was significantly associated with the perception of taste pleasantness [F_(4,56)_ = 3.721, *p* < 0.05], and the model explained around 22% of the variance (Table 4A). In patients with hyposmia, the AWV odor intensity was significantly associated with taste intensity [F_(2,28)_ = 7.362, *p* < 0.05], and the model explained around 29% of the variance (Table 4B).

## 4. Discussion

The comprehension of sour taste physiology shows that it plays an important role in the flavor perception of acid foods. Acids are found in many foods, beverages, jams, fats, and oils; their main use is in the enhancement of food and beverage flavors.

First, we assessed differences in sourness perception in patients with pure hyposmia and those with a neurodegenerative disorder (PD) compared to healthy controls. Our data indicated that subjects with pure hyposmia and those with PD showed significantly a higher mean age than subjects with normosmia. This finding could be explained considering that aging, a progressive physiological degeneration of all functions, is associated with deficits in olfactory and gustatory systems. However, aging differently affects the taste perception, involving diverse mechanisms for each taste modality. Sourness perception usually decreases during aging, as indicated in previous studies [39,40]. Our data indicated that in patients with pure hyposmia, the decrease in gustatory function varied between taste modalities, since only in sour taste perception was there observed a significant decrease compared to healthy controls. A previous study showed that older adults exhibited a decline only for salty and sour taste perception, and no differences were found for sweet and bitter perception [41]. This finding could be explained considering different brain activations. In older adults, lower brain activation was observed in inferotemporal regions in response to sour stimuli compared to younger subjects [40]. The inferotemporal regions play an important role in homeostasis and appetite regulation, and selectively modulate an individual’s preference to maintain homeostasis [42]. In older adults, a decrease in sour taste perception was observed when the acidity in body tissue increased [43].

Instead, in PD patients olfactory and gustatory dysfunctions were related to cognitive ones, as indicated in previous studies [44,45,46,47,48]. PD patients showed a significant decrease in global olfactory and gustatory function compared to healthy controls. Olfactory and gustatory dysfunction in PD are connected to different anatomical pathways. As indicated in previous studies, olfactory deficits usually precede the appearance of motor symptoms in PD and are reported in over 96% of patients [49,50,51]. Olfactory dysfunction has been considered as a supportive criterion in clinical PD diagnosis [51].

Our data indicated severe impairment in OT, OD, OI, and TDI scores in PD patients compared to those with hyposmia and to healthy controls. These data suggest a central and peripheral impairment in PD patients, since OT is more associated with sensorial processing, which mainly depends on the peripheral and subcortical part of the olfactory system. In contrast, OI and OD are the ability to identify and differentiate between odorants, respectively, [52] and activate central brain areas. The potential causes of olfactory dysfunction are not well understood, although α-synuclein deposition has been identified in the olfactory bulb, anterior olfactory nucleus, and olfactory cortex of PD patients [49,50,51].

Moreover, our data showed that PD patients exhibited a significant decrease in sweet, salty, sour, and bitter taste perception compared to those with hyposmia and to healthy controls. Gustatory dysfunction has been associated with advanced stages of the disease, with a dysregulation of the TAS1R and TAS2R families. Our data suggested a decline in each taste modality, with different biological effects, as previously reported [46,47,53]. Sweet and salty taste are related to high nutritional foods, and their impairment may lead to metabolic/cardiovascular disease and obesity [54], while sour and bitter may reflect damage in the detection of dangerous foods [55].

The decrease in gustatory and olfactory functions during aging is associated with decreased enjoyment in food intake, poor nutrition, and altered eating behavior, with a negative impact on human life [56,57,58,59]. The comprehension of sourness perception may help the development of specifically enhanced foods for older adults to compensate for chemosensory deficits. Aromatic herbs and spices may represent a strategy as flavor enhancements in dietary use [26,27,60]. The use of herbs and spices may play a key role in the modulation of salty [26,27] and bitter taste perception [27,29]. In the Mediterranean basin, white wine vinegar is amply used as condiment and flavor enhancer. White wine vinegar is traditionally obtained through the spontaneous acetification of wine conducted by acetic acid bacteria [61].

Then, in our study, in a subpopulation of patients with pure hyposmia, we assessed the effect of myrtle aromatization on the sensory characteristics and acceptability of white wine vinegar. The sensory perception of a traditional white wine vinegar and a white wine vinegar aromatized with myrtle were evaluated to find the potential role of this aromatic plant. Our data suggested that myrtle aromatization increased the number of significant correlations between odor and taste dimensions of the white wine vinegar both in controls and in patients with hyposmia in a different manner. These correlations may be explained by considering the complex multisensory interaction between the retronasal perception of myrtle aromatic volatile compounds in the oral cavity responsible for the flavor attributes, the taste perception on the taste buds of myrtle non-volatile polar bitter/astringent components, and the modulation of the trigeminal pathway.

The potential role of myrtle aromatization may be to modulate the white wine vinegar odor and taste dimensions in a different manner in controls and in patients with hyposmia. In controls, myrtle aromatization odor intensity was significantly associated with the perception of taste pleasantness, while in patients with hyposmia, the AWV odor intensity was significantly associated only with the taste intensity.

Our study suggested large individual variability in vinegar odor and taste intensity, both in controls and patients with hyposmia. This large individual variability may be related to changes in taste papillae morphology and number but also to genetic and environmental factors, as indicated in previous studies for other chemosensory perceptions [5,62,63]. A large individual variability was also observed in the human oral response to bitter stimuli and capsaicin, as indicated in previous studies [64,65]. Moreover, the odor and taste pleasantness of vinegar may be related to familiarity and frequency of consumption [66].

Myrtle aromatization may modulate the perceptions of acetic acid in white vinegar through a reduction in the nasal and oral irritation induced by volatile and non-volatile compounds, respectively. The nasal and oral irritation are usually described as trigeminal stimuli mediated by the trigeminal nerve in the oral cavity.

Previous studies indicated intimate correlations between the olfactory function and trigeminal system [67,68]. In patients with hyposmia, no significant differences were observed for mean values of WV odor and taste intensity compared to controls. This result suggests a decrease in olfactory function (hyposmia) associated with the presence of a compensatory increase in trigeminal function. In the central nervous system, an olfactory deficit is associated with a reduced trigeminal perception due to a decrease in central nervous system interactions. However, at the peripheral level, an olfactory deficit induces the adaptative mechanism, with an increase in trigeminal response in patients with hyposmia [68].

## 5. Conclusions

In this study, we evaluated sourness perception in patients with hyposmia compared to controls. Our data indicated that in patients with hyposmia, the decrease in gustatory function varied between taste modalities, since only in sour taste perception was there observed a significant decrease compared to healthy controls. Then, in our study, we assessed the effect of myrtle aromatization on the sensory perception and acceptability of sour food. The sensory perception of a traditional white wine vinegar and a white wine vinegar aromatized with myrtle was evaluated to find the potential role of this aromatic plant. Our data suggested that myrtle aromatization increased the number of significant correlations between the odor and taste dimensions of the WV both in controls and in patients with hyposmia in a different manner. The increase in vinegar aroma due to myrtle aromatization modulated sourness perception in patients with hyposmia. These data confirmed the important role of aromatic herbs and spices in the enhancement of chemosensory perception in patients with olfactory deficits.

## Figures and Tables

**Table 1:** Digital images and composition of the two types of commercial white wine vinegars.

Sample		Nutritional Values (Per 100 mL of Product)	Acidity
White wine vinegar (WV)	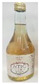	Proteins 0.1 g; Carbohydrates 0.05 g (Sugars 0 g); Fat 0 g (Saturated 0 g); Sodium 5 mg; Energy value 21 kcal/88 kJ	6%
White wine vinegar aromatized with myrtle (AWV)	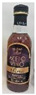	Proteins 0.1 g; Carbohydrates 0.05 g (Sugars 0 g); Fats 0 g (Saturated 0 g); Sodium 5 mg; Energy value 21 kcal/88 kJ	6%

**Table 2:** Demographic and clinical parameters of all subjects enrolled in the study. Results are expressed as mean values ± standard deviation.

Parameters	Controls	Hyposmia	PD
Age	33.47 ± 13.33	36.29 ± 16.34 ***	69.07 ± 9.88 *** ^§§§^
Weight	62.73 ± 13.35	65.81 ± 16.29 **	72.40 ± 15.89 *** ^§§§^
Height	1.64 ± 0.08	1.65 ± 0.09	1.65 ± 0.09
BMI	23.14 ± 4.21	24.34 ± 7.41	26.56 ± 4.84 * ^§§^
MoCA	27.94 ± 1.67	27.41 ± 2.13	20.40 ± 6.42 *** ^§§^

Legend: Montreal Cognitive Assessment (MoCA) Scale. Statistical differences were established by one-way ANOVA followed by Bonferroni’s post hoc test; *** = *p* < 0.001, ** = *p* < 0.01, * = *p* < 0.05 for patients with hyposmia versus those with PD versus controls; ^§§§^ = *p* < 0.001, ^§§^ = *p* < 0.01 between patients with hyposmia and those with PD.

**Table 3:** Demographic and clinical parameters of patients with hyposmia (*n* = 39) compared to controls (*n* = 57). Results are expressed as mean values ± standard deviation.

Parameters	Controls	Hyposmia	Significance
Age	34.30 ± 15.69	34.62 ± 15.18	*p* > 0.05
Weight	60.28 ± 11.41	62.85 ± 16.78	*p* > 0.05
Height	1.64 ± 0.08	1.65 ± 0.09	*p* > 0.05
BMI	22.28 ± 3.52	22.98 ± 4.68	*p* > 0.05

**Table 4:** Multiple linear regression analysis performed for odor and taste dimensions (pleasantness, intensity, and familiarity) of aromatized white wine vinegar (AWV) in healthy controls (*n* = 57) (A) and patients with hyposmia (*n* = 39) (B).

Parameters	Unstandardized Coefficients		Standardized Coefficients		
	B	Std Error	β	t	*p*
AWVOI as a dependent variable					
(A) CONTROLS					
AWVOF	0.139	0.189	0.166	0.734	0.466
AWVTP	0.223	0.097	0.296	2.295	0.026 *
AWVTI	0.256	0.133	0.239	1.928	0.059
AWVTF	0.011	0.187	0.013	0.056	0.955
(B) HYPOSMIA					
AWVOF	0.133	0.091	0.223	1.465	0.152
AWVTI	0.326	0.120	0.412	2.710	0.010 *

Legend: AWVOI = aromatized white wine vinegar odor intensity; AWVOF = aromatized white wine vinegar odor familiarity; AWVTP = aromatized white wine vinegar taste pleasantness; AWVTI = aromatized white wine vinegar taste intensity; AWVTF = aromatized white wine vinegar taste familiarity. * = *p* < 0.05.

## Data Availability

The datasets generated and analyzed during the current study are available from the corresponding author on reasonable request.
